# Recurrent Palatal Pleomorphic Adenoma: A Case Report With a Long-Term Follow-Up

**DOI:** 10.7759/cureus.26363

**Published:** 2022-06-27

**Authors:** Hemavathy K, Giri G V V, Vijayanirmala Subramani, Susruthan M

**Affiliations:** 1 Oral and Maxillofacial Surgery, Sri Ramachandra Institute of Higher Education and Research, Chennai, IND; 2 Oral and Maxillofacial Pathology, Sri Ramachandra Institute of Higher Education and Research, Chennai, IND; 3 Pathology, Sri Ramachandra Institute of Higher Education and Research, Chennai, IND

**Keywords:** revision of surgery, recurrent tumor, p40, palate, minor salivary gland

## Abstract

Pleomorphic adenoma is the most common kind of major tumor of the major and minor salivary organs. Although pleomorphic adenoma is a benign tumor, it has a high chance of recurrence and malignancy. In the literature, lower rates of repetitive pleomorphic adenoma of the sense of taste have been detailed while the palate is a common location for an intraoral pleomorphic adenoma. Recurring tumors have been associated with a high risk of malignancy, and surgical excision is the basic treatment option for recurrent adenomas. Revision surgery is quite challenging and has never been standardized. We report a rare case of recurrent pleomorphic adenoma of the palate that occurred 7.5 years after primary ablation.

## Introduction

Recurrent pleomorphic adenoma can present decades after resection of the primary tumor. The clinical presentation of patients with recurrent pleomorphic adenoma can be with a uninodular/isolated or solitary lump, multinodular, extending above and below the scar or surgical bed [1], or bone destruction-mimicking malignant lesions. The incidence rate of recurrence has been associated with histopathological variants and genetic characteristics [2]. The spectrum of imaging findings in recurrent lesions ranges from homogenous solid to heterogeneous rim-enhancement patterns. The recommended treatment for recurrent pleomorphic adenoma includes surgical excision, radiation therapy, or a combination of these [3].

## Case presentation

**Figure 1:**
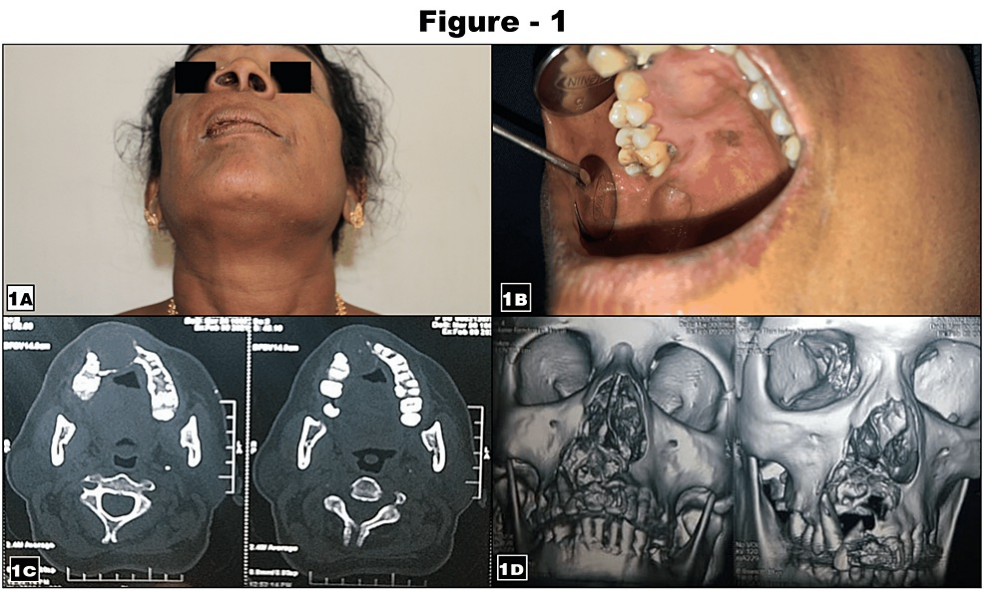
Preoperative clinical presentation, computerized tomography (CT), and three-dimensional imaging of recurrent pleomorphic adenoma 1A. Shows facial asymmetry in the upper anterior region, 1B. Shows intra-oral swelling in the anterior maxilla, 1C. CT imaging shows an osteolytic lesion evident on the right maxilla, and 1D. 3D imaging shows a destructive lesion

A 58-year-old female patient presented with a painless mass and a mild facial deformity in the right anterior maxilla for the past four months (Figure 1A). The patient had been operated on for pleomorphic adenoma of the hard palate on the right side seven years ago. Physical examination demonstrated a 3X2 cm, well-circumscribed, smooth, firm, non-tender, fixed swelling over the anterior maxilla (Figure 1B) and there were no palpable lymph nodes. The differential diagnosis included a palatal abscess, odontogenic or non-odontogenic cyst, soft tissue tumors, or recurrent lesions. Computerized tomography (CT) imaging demonstrated cystic appearing nodules with peripheral enhancement. We also performed 3D reconstruction imaging, which revealed a tumor mass involving the pyriform rim, crossing midline, and extending posteriorly, involving 14 tooth regions (Figures 1C-1D].

**Figure 2:**
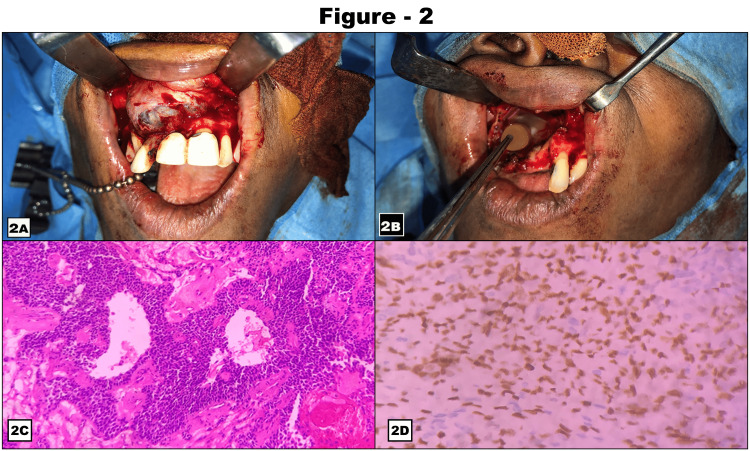
Intraoperative clinical presentation; microscopic features of lesions 2A & 2B show lesion tissues on the table and 2C & 2D depict small cells arranged in a ductal pattern and P40 immunoreactivity positive for myoepithelial cells.

**Figure 3:**
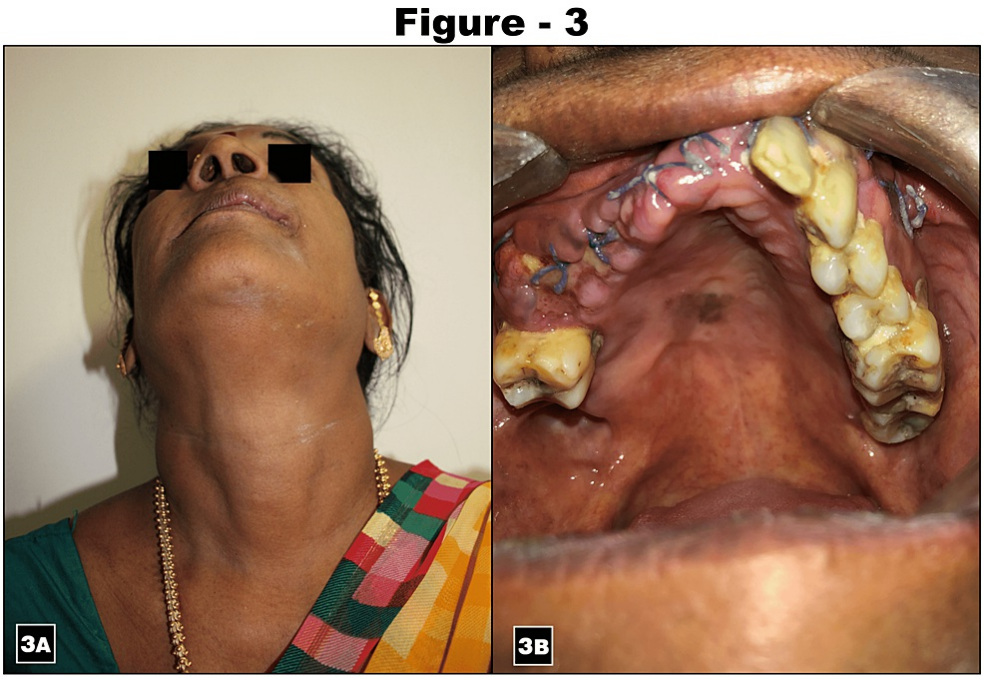
Postoperative follow-up clinical pictures 3A & 3B show extra and intra-oral clinical presentation

Fine-needle aspiration biopsy (FNAB) of the mass revealed plenty of RBCs with scanty cells. The routine investigation included a chest X-ray and biochemistry, and a hemogram was required for surgical fitness. A treatment plan for the excision of the lesion in the anterior maxilla was formulated. A crevicular incision was placed from the 23 to 15 tooth region with a vertical releasing incision placed distal to the 15 and 23 teeth. The mucoperiosteal flap was elevated. A cyst related to the anterior maxilla was identified, and cyst enucleation was done. Extraction of teeth number 11, 12, 13, 14, 15, and 21 and contouring of alveolar bone was done. Chemical cauterization of cystic lining was performed using Carnoy's solution. Saline irrigation was also done. Platelet-rich plasma (PRP) was placed in the cystic cavity and closure was done using 3-0 Vicryl (Figures 2A-2B). Throughout the procedure, multiple specimens were collected for intraoperative frozen sections. All specimens were interpreted as favoring a benign odontogenic tumor - adamantinoma. The remainder of the specimen was examined following standard histological processing and staining with hematoxylin and eosin (H&E). The H&E section (Figure 2C) showed a tumor composed of small cells with a moderate amount of cytoplasm favoring glomangioma. Immunohistochemistry analysis was performed with a panel of markers - vimentin, cytokeratin (CK), S100, SMA, Ki67, and P40. Vimentin was diffusely positive, S100 was focally positive, SMA was negative, and P40 was positive in myoepithelial cells (Figure 2D). CK was positive in epithelial cells Ki67 - 5%; this staining pattern favors the diagnosis of recurrent pleomorphic adenoma. The patient had an uneventful postoperative recovery and follow of 10 months revealed no re-recurrence (Figure 3). 

## Discussion

Recurrent pleomorphic adenoma of the palate is uncommon and usually presents as a diffuse multifocal mass at the primary site or distant from the primary site. Fewer solitary nodule recurrence types were reported, and the exact cause of pleomorphic adenoma recurrence remains elusive. In most patients, recurrence lesions present at a time interval of seven to 10 yrs after the initial surgery. Two incidences of recurrence of pleomorphic adenoma of the palate in the child were reported [4]. Signs and symptoms are nonspecific and size varies from 0.5-5 cm [3]. In the assessment of recurrent disease, imaging like MRI and CT provides the size of the tumor, erosion, and perforation or infiltration into the palate bone and cranial cavity. A massive recurrent pleomorphic adenoma of the palate involving the cranial base was reported by Faisal [5]. A present case report showed a 58-year-old female patient with recurrent pleomorphic adenoma of the palate, primary has been operated on seven years ago. This was consistent with literature that a peak incidence of fifth decades of life.

The imaging of recurrent tumor is similar to that malignant process and should be conscious of the possibility of malignant change in recurrent intraoral pleomorphic adenoma. The diagnostic workup of histopathological findings along with immunohistochemistry was a useful tool for the final diagnosis of the present case. De Lima FF et al. stated that the recurrent pleomorphic adenoma arising in the oral cavity is suitable for further surgical resection of the involved tissues with reconstruction by a pedicle or free tissue transfer with or without bone replacement and these procedures are followed by radiotherapy [6]. External beam and neutron radiotherapy may be alternative treatments offered to select patients. Frequent or persistent pleomorphic adenoma recurrences may proceed to metastasis with a potentially fatal outcome [7-8].

## Conclusions

The recurrent palatal pleomorphic adenoma is rare because, due to the minor salivary gland, these tumors have little or no capsule. At the same time, these tumors are challenging entities to diagnose and treat. Palatal cases of recurrent pleomorphic adenoma are large and solitary, well-delineated, with smooth margins. Recurrent tumors cause bone destruction by mimicking malignant lesions. Most imaging studies guide the clinical presentation of solitary or multiple nodules adjacent to the operative bed and should suggest the diagnosis of recurrence. In our case report, we found the mucous subtype of palatal pleomorphic adenoma has an incidence of recurrence. The present case report emphasizes cystic appearance; more prospective investigations like imaging techniques, along with immune profiling, will be essential for the accurate diagnosis of recurrent lesions.
